# Determinants of Trajectories of Informal Caregiving in later life. Evidence from England

**DOI:** 10.21203/rs.3.rs-4027872/v1

**Published:** 2024-04-29

**Authors:** Giorgio Di Gessa, Christian Deindl

## Abstract

Although long-term consequences of informal care provision are well investigated, fewer studies have examined trajectories of informal care provision among older people and the socioeconomic, demographic, health, and family characteristics associated with them. We use data from four waves of the English Longitudinal Study of Ageing, with 6,561 respondents followed for 6 years (2012/3 to 2018/9). We used group-based trajectory modelling to group people's provision of care over time into a finite number of distinct trajectories of caregiving. Using multinomial logistic regressions, we then investigated characteristics associated with these trajectories. Four distinct trajectories were identified representing “stable intensive”, “increasing intensive”, “decreasing”, and “stable no care”. Results suggest that, although there are socioeconomic, demographic, and health differences across the trajectories of caregiving (with younger women in good health and poorer socioeconomic status more likely to care intensively throughout), family characteristics are their main drivers. Respondents who live alone, with no children, and no parents alive are more likely to never provide care, whereas those with older parents and who live with adults in poor health are more likely to provide stable intensive care. Also, changes in family characteristics (e.g. death of parents, widowhood, or deterioration of the partner’s health) are associated with trajectories representing increases or decreases of caregiving over time. Overall, trajectories of informal caregiving undertaken by older people are varied and these patterns are mostly associated with both the availability and health of family members, suggesting that the needs factors represent the most immediate reason for caregiving commitments.

### 1. Introduction

Informal caregivers provide unpaid care, often to family members or friends in need of support and care due to long-term physical disability, mental health condition, or chronic disease. While the prevalence of informal caregiving depends on the definition used, recent estimates suggest that about 17% of the adult population in Europe provide informal care (Tur-Sinai et al. 2020), with those in late mid-adulthood (aged 50 to 64) often most likely to become informal caregivers and to make up the majority of carers (Lacey et al. 2024; Larkin et al. 2019; ONS 2023). Moreover, many caregivers provide several years of care; according to the UK Office for National Statistics, men and women aged 50 can expect to spend 4.9 and 5.9 years of their remaining life as unpaid carers (ONS 2017).

Although there is growing interest in caregiving research in later life and an increasing body of research exploits available longitudinal datasets, to date most of these studies have focused on the consequences and sequelae of care provision (Larkin et al. 2019; Schulz et al. 2020). There is extensive evidence about the (generally negative) impact of care provision on carers’ mental and physical health, employment and finances, as well as social life (Bauer and Sousa-Poza 2015; Bom et al. 2018; Keating and Eales 2017; OECD 2011; Price and Di Gessa 2023), although it is acknowledged that links between caregiving and carer outcomes vary by socioeconomic and demographic characteristics, the extent of and involvement in providing care, as well as the clinical conditions of and the relationship with the care recipients (Brown and Brown 2014; Zueras and Grundy 2024).

Longitudinal studies have often also investigated patterns of caregiving received (rather than *provided*) by older people and have shown how increases or reductions in the amount of care received by older people over time often occurred in response to changes in people's functional status and the needs of the individuals (Deeg et al. 2005; Miller and McFall 1991) as well as the availability and provision of public-funded formal care (Kjær and Siren 2020; Li 2005). Although there is considerable heterogeneity in older people’s trajectories of frequency and intensity of care utilisation (Hu 2020), care receipt often increases over time after the disease onset, particularly for those who suffer from degenerative conditions that require more assistance over time (Jutkowitz et al. 2020).

To date, however, few studies have analysed longitudinal patterns of caregiving despite informal caregiving being often conceptualised as a dynamic process wherein several changes may occur (Schulz et al. 2020; Uccheddu et al. 2019; Verbakel and Glijn 2023). Indeed, depending on the care recipient’s functioning and needs and their relationship with the carer, care provision might involve different tasks and complexity of activities (from practical and emotional support to medical and self-care help), different frequency and levels of intensity (from around-the-clock daily to sporadic one-off hours of care), and different progressions (that could increase, decrease, or even end over time depending on how critical and long-lasting is the illness or need of the person cared for). Therefore, it is important to describe longitudinal trajectories of informal caregiving to better understand the dynamic nature of informal care in later life as well as to examine the characteristics and family situations of those experiencing different trajectories of caregiving.

To our knowledge, studies on ‘trajectories of informal caregiving’ have mostly used two time points to capture changes in informal care provision. For instance, drawing data from two longitudinal studies conducted in Massachusetts during the 1970s and 1980s, Jette and colleagues’ study (1992) was one of the first to examine stability and changes in caregiving patterns. Since then, several other studies have investigated these patterns of care but have done so mostly considering two time points, and therefore distinguishing between broad categories of “continuing”, “starting”, “stopping”, and “never” caregiving patterns (Lawton et al. 2000; Lee and Gramotnev 2007; McCann et al. 2004; Robards et al. 2015). Among the noticeable exceptions, Tooth and Mishra (2014) used data from the Australian Longitudinal Study on Women’s Health collected over 9–13 years (i.e. 4 to 5 waves of data) to identify trajectories of care provision. Among older cohort members, the authors identified three classes that distinguished between women with constantly low or high probabilities of being a carer and a third group of women who initially provided no care to then show a substantial increase in caregiving. The authors also identified demographic, socioeconomic, and health characteristics associated with these trajectories of caregiving, showing that women with relatively poorer socioeconomic background were more likely to provide continuing care throughout the 9-year period under study. Using retrospective data from a panel sample of Dutch carers aged 16 to 78, Verbakel and Glijn (2023) also showed heterogeneous trajectories of caregiving and identified three classes of care that represented a decrease, stability, and increase in care demands (assessed with care receivers’ health condition), intensity of care (i.e. number of informal caregiving hours and duration of care episodes), and care complexity (captured by number and types of caregiving tasks). The authors also found that different trajectories were related to both the age and the living arrangement of the care receiver. Although both studies offer important contributions to the literature, namely highlighting how care provision changes over time, still little is known about how and to what extent trajectories of informal caregiving relate to the (changing) ability of the informal caregivers to provide care and happen in response to (changing) family situations and their potential needs of care.

In this paper, therefore, first, we describe, at a population level, patterns of informal caregiving over time among older people in England, moving beyond "snapshots" that are often used to describe care provision in later life and accounting for the intensity of care provided (Keating et al. 2019). Second, to examine factors underpinning variations in care provision, we analyse which individual and family characteristics are associated with distinct trajectories of informal caregiving in later life, investigating both changes in the caregiver as well as in their family situations (including death of partners or spouse, or deterioration of a partner's health). In our analyses, we will be as inclusive as possible and consider associative relationships between a wide range of factors and trajectories of informal caregiving to best understand the profiles of those whose commitment to care provision remains intense or decreases/increases over time (and possibly identify those at higher risk or increasing care provision). Taken together, our study aims to provide valuable additional insights into the dynamic nature of care provision in later life.

### 2. Methods

### 2.1 Study Design and Participants

Data were obtained from the English Longitudinal Study of Ageing (ELSA). This is an ongoing multidisciplinary longitudinal nationally representative survey of individuals aged 50 and older who live in private households in England (Banks et al. 2021). Specific details of sampling frames and methodology, weighting strategies and questionnaires can be found at www.elsa-project.ac.uk. ELSA started in 2002 and data are collected biennially using face-to-face personal interviews and self-completion questionnaires, with the most recent full wave of data collection in 2018–19 (wave 9). Informed consent was obtained from all participants. All data are available through the UK Data Service (SN 5050).

Our sample consisted of non-proxy participants who had been successfully interviewed between Waves 6 (2012/13) and 9 (2018/9) and with available information about care provision (the main variable of interest) in at least one wave. We did not include previous waves because the questions on informal care provision were asked differently and/or had different filters/routings. The final analytical sample consisted of 6,561 ELSA participants (94% of which were present in all four waves under study).

### 2.2 Main Measurements of Interest

**Outcome**

In Waves 6–9 of ELSA, all respondents were asked two questions about the provision of care. In the first, respondents were asked (in the "Work and Pensions" module) if among the activities done in the previous month they also "cared for someone". They were then also asked whether they "looked after anyone in the past week". Those who looked after someone in the week before the interview were then asked a series of follow-up questions including who they looked after, how many hours, how many people they cared for, and whether any of the care recipients lived with them. For the main variable of interest, respondents were then classified as "not providing care", "providing intensive care" if they looked after someone they lived with or someone outside of the household for more than 10 hours in the previous week, and "providing non-intensive care" if they cared for someone living outside of the household for fewer than 10 hours in the previous week or if they did so in the previous month only.

**Covariates**

In line with the informal care model (Broese van Groenou and De Boer 2016), we accounted for a wide range of demographic; socioeconomic; health; and social relationships covariates. All covariates were assessed at Wave 6, hereafter also referred to as "baseline" measurements. As demographic factors we considered gender and age modelled as a categorical variable, distinguishing those aged 50–59, 60–69, and 70 and older. Socioeconomic factors included education, wealth, employment, and volunteering. Educational level was recoded into a binary variable distinguishing between low (below secondary) and middle/high education following the International Standard Classification of Education (http://www.uis.unesco.org/). Wealth was equal to the total net non-pension non-housing wealth, and respondents were categorised into wealth tertiles. For employment status, we classified respondents as being in paid work or not. Finally, respondents reported if they had volunteered in the month before the interview.

Health variables included self-perceived health, physical disability, depression, and multimorbidity. Self-rated health (SRH) was measured on a five-point ordinal scale (excellent, very good, good, fair, or poor). The five SRH items were dichotomised into "fair or poor" versus better health. Physical disability was assessed using limitations in activities of daily living (ADL, such as getting out of bed and walking across a room) and instrumental ADL (such as shopping for groceries and preparing a hot meal). Participants who reported limitations with one or more activities were defined as having a physical disability. For mental health, ELSA included an abbreviated eight-item version of the Centre for Epidemiologic Studies Depression Scale (CES-D) (Radloff 1977). Respondents are asked whether they had experienced any depressive symptoms, such as restless sleep or being unhappy, in the week before the interview, with those reporting four or more classified as 'depressed' (Steffick 2000). Finally, we classified respondents as having multimorbidity if they reported two or more long-term medical conditions (including high blood pressure, coronary heart disease, stroke, diabetes, and cancer) (Zaninotto et al. 2020).

Among indicators of family composition, we considered the presence of potential dependents who might require care as well as their health, where possible. In particular, we included indicators of whether respondents have any living siblings or not, as well as whether they have any children, and among those with children whether at least one child lives with the respondent. For (biological) parents, ELSA does not collect information on their health; therefore, considering parents' age as a crude health proxy, we classified respondents as having no parents alive, parents younger than 85, or at least one parent aged 85 or older. Moreover, ELSA collects information on all consenting adults aged 50 + and respondent's partners (regardless of their age). Exploiting the study design and using both the health indicators described above and the household composition, we constructed a variable that not only accounted for whether the respondents lived with other respondents aged 50 and older (mostly their partners) but also whether that person was in overall good health.

**Changes over time**

As the covariates mentioned above were assessed in all ELSA waves, we also considered most of the social relationships and health indicators in terms of changes over the 6-year follow-up period. Depending on the variables and their distributions, we created variables capturing changes over time or disruptive events. For instance, for health-related variables, we considered categories such as "no change", "health has improved" and "health has deteriorated"; whereas for social indicators we constructed binary indicators capturing widowhood or death of parent(s).

**Statistical Analysis**

First, the percentages of respondents who provide care were calculated at each wave under study. In order to identify distinctive trajectory patterns of informal care provision, we used group-based trajectory modelling (Nagin 1999; Nagin and Odgers 2010); this method is used to cluster individuals into meaningful subgroups, each with a similar underlying trajectory of caregiving. This method takes into account the dependency of observations and assumes a mixture of subpopulations with different individual trajectories within the target population and identifies distinctive groups within which individuals share similar developmental trajectories (Herle et al. 2020; Nguena Nguefack et al. 2020). To determine the number of trajectory groups within our sample, we fit a series of group-based trajectory models with up to six groups. Missing data were handled using full information maximum likelihood estimation. In selecting the appropriate number of trajectory groups, we considered a wide range of criteria including the Akaike Information Criterion (AIC) and the Bayesian Information Criterion (BIC). For each of these, lower scores indicate (relatively) better fitting models. Moreover, we additionally considered the average posterior probabilities of group membership as a measure of classification quality; group size (and the avoidance of too small classes that may lead to a lack of reproducibility of the results); the usefulness of the number of groups in terms of the similarities/differences in their trajectories; and the interpretability of the distinctive trajectories (Nagin and Odgers 2010; Nguena Nguefack et al. 2020).

Once the trajectories were identified, we first examined the (un-adjusted) differences among these trajectory groups in terms of demographic; socioeconomic; family; and health-related covariates at baseline (using chi-squared tests). Second, we used multinomial logistic regression analyses to examine the combined effects of these characteristics on respondents' group membership of different trajectories of informal caregiving. Third, we examined the associations between trajectories of caregiving and changes in selected social relationships and health indicators (controlling for baseline basic sociodemographic characteristics). To ease the interpretation of results, the findings are reported as average marginal effects (AMEs) for the explanatory variable. Due to the categorical nature of our outcomes and explanatory variables, the AMEs are to be interpreted as the discrete effect of the independent variable (compared to the reference category), i.e., as the difference between the predicted probabilities (in percentage points) across the groups being compared. Trajectories were determined using Mplus; data management and statistical analyses were performed using Stata 18.

### 3. Results

## 3.1 Distinctive trajectories of informal caregiving

Table 1 shows the distribution of the longitudinal care provision categories across all four waves under study. Overall, each of the three categories of care provision calculated for ELSA participants shows stable probabilities over time. The majority of respondents (about three in four) are classified as not providing any care across all waves, with about 11/12% of respondents reporting intensive care at each wave.

To summarise the dynamic process of informal care provision over time and determine the optimal number of trajectory groups, a series of group-based trajectory models were fitted (with a specification of up to six trajectory groups). Based on the goodness-of-fit criteria (shown in Supplementary Table 1) and the other considerations mentioned above, we identified four as the number of trajectories that best fit the data. The cumulative predicted probabilities of each of the three categories varied substantially across classes (Fig. 1). Class 1 (4.9%) and Class 3 (65.1%) identify groups of respondents who report respectively "intensive informal care" and "no informal care" with high and fairly stable probabilities. Respondents in these groups are hereafter classified as "*stable intensive care*" and "*stable no care*" respectively. The other two classes show time-varying probabilities: Class 2 (6.9%) comprises older people who initially do not provide informal care but progressively look after people and do so more intensively over time ("*increasing intensive care*") while Class 4 (23.1%) includes those whose probabilities to be informal caregivers (roughly equally split between intensive and non-intensive) steadily decreases over time ("*decreasing care*").

Table 2 shows the characteristics of the care provided in the week before the interview at each Wave by those whose probabilities of reporting care in their trajectories are not null (that is, all respondents but those who were clustered in the "*stable no care*" group). Overall, as one would expect, respondents in the "*stable intensive care*" report high average hours of care per week, with about one third providing care 24/7. About three in four of those classified as "stable intensive carers" also live with the person they care for, that is in the majority of the cases their partner/spouse. Across the waves under study, the intensity of care provided by this group increases, and these respondents become more likely to care for just their partners. Those in the "*decreasing care*" group, on the other hand, experience a reduction of their overall commitment to this task; these informal carers are most likely to care for friends and "other" family members and over time a smaller percentage care for their parents/parents-in-law. Finally, the "*increasing intensive care"* providers, after a relatively modest initial engagement in this activity, spend increasingly more time looking after people and are more likely to mention that they care for someone they live with, their spouse, or parent(s)/in-law.

Table 1
Summary of longitudinal data on care provision

| | N | No care | Non-intensive care | Intensive care |
|---|---|---|---|---|
| **Wave 6** | 6,547 | 4,931 (75.3%) | 783 (12.0%) | 833 (12.7%) |
| **Wave 7** | 6,300 | 4,805 (76.3%) | 797 (12.6%) | 698 (11.1%) |
| **Wave 8** | 6,221 | 4,773 (76.7%) | 697 (11.2%) | 751 (12.1%) |
| **Wave 9** | 6,336 | 4,901 (77.4%) | 707 (11.2%) | 728 (11.4%) |

Source: English Longitudinal Study of Ageing (ELSA) Waves 6 (2012/13) – 9 (2018/19).

Legend:

Notes: These probabilities are predicted by the best-fitting group-based trajectory model with 4 classes.

Source: English Longitudinal Study of Ageing (ELSA) Waves 6 (2012/13) – 9 (2018/19). N = 6,561

Table 2
Caregiving characteristics by trajectories of informal caregiving and waves

| | | Wave 6 | Wave 7 | Wave 8 | Wave 9 |
|---|---|---|---|---|---|
| **Mean number of hours of care provided** | *Stable intensive care* | 67.5 | 66.6 | 71.9 | 81.8 |
| | *Increasing intensive care* | – | 5.1 | 37.3 | 45.0 |
| | *Decreasing care* | 32.6 | 32.8 | 28.6 | 26.0 |
| **% Caregiving 24 hours a week** | *Stable intensive care* | 32.6 | 32.0 | 34.8 | 40.8 |
| | *Increasing intensive care* | – | 2.0 | 15.5 | 20.0 |
| | *Decreasing care* | 12.6 | 12.9 | 10.6 | 9.5 |
| **% Living with the person they cared for** | *Stable intensive care* | 72.7 | 74.1 | 79.6 | 80.5 |
| | *Increasing intensive care* | – | 2.9 | 46.4 | 48.4 |
| | *Decreasing care* | 33.5 | 33.3 | 31.1 | 25.3 |
| **% Caregiving for 2 or more people** | *Stable intensive care* | 29.1 | 25.1 | 20.8 | 19.1 |
| | *Increasing intensive care* | – | 37.1 | 28.3 | 26.9 |
| | *Decreasing care* | 32.2 | 28.0 | 26.3 | 30.2 |
| **% Caregiving for spouse/ partner** | *Stable intensive care* | 56.4 | 59.9 | 63.3 | 64.1 |
| | *Increasing intensive care* | – | 2.9 | 39.2 | 42.3 |
| | *Decreasing care* | 26.2 | 26.4 | 25.3 | 23.4 |
| **% Caregiving for parents/ in-laws** | *Stable intensive care* | 19.4 | 17.8 | 15.5 | 12.7 |
| | *Increasing intensive care* | – | 14.1 | 17.9 | 20.5 |
| | *Decreasing care* | 24.6 | 21.3 | 17.7 | 17.1 |
| **% Caregiving for a child** | *Stable intensive care* | 14.5 | 12.6 | 11.7 | 13.9 |
| | *Increasing intensive care* | – | 0.0 | 4.6 | 4.6 |
| | *Decreasing care* | 7.2 | 5.4 | 7.6 | 6.5 |
| **Caregiving for other family/ friends** | *Stable intensive care* | 24.5 | 23.1 | 18.7 | 18.4 |
| | *Increasing intensive care* | – | 30.0 | 30.4 | 30.7 |
| | *Decreasing care* | 39.0 | 35.2 | 34.4 | 36.2 |

Source: English Longitudinal Study of Ageing (ELSA) Waves 6 (2012/13) – 9 (2018/19). N = 6,561.

Notes: Caregiving characteristics refer to those who provided care for someone in the week before the interview and are not reported for respondents whose group-based trajectory class presented a relatively high probability of not providing care throughout the Waves under study ("*stable no care*"). Percentages do not necessarily add to 100 as some respondents cared for two or more people. Values at Wave 6 for the "*increasing intensive care*" groups are not reported (–) because they are based on less than 30 respondents.

### 3.2 Determinants of trajectories of informal caregiving

Table 3 summarises ELSA respondents' baseline characteristics and shows the distributions of their demographic, socioeconomic, health, and family characteristics among the four caregiving trajectory groups. Overall, men and people aged 70 and older are more likely to be in the "stable no care group". Generally speaking, those who cared intensively over time had the lowest levels of wealth and engagement in both paid and voluntary work. When health is considered, those in the "stable intensive care" group were more likely to report poor health; whereas respondents in the "increasing intensive care" group (who initially are not engaged in caregiving activities) had the best health profile at baseline. Finally, as expected, respondents in the "stable no care" group were more likely to have no children, no parents alive, and to live on their own. Among those who live with other people, "stable intensive" carers were overwhelmingly likely to reside with adults (mostly their partners) in poor health.

Table 3
Demographic, socioeconomic, health, and family characteristics of the ELSA sample by caregiving trajectories

| | *Stable intensive care* | *Increasing intensive care* | *Stable no care* | *Decreasing care* | *Total* | P value |
|---|---|---|---|---|---|---|
| Female | 63.4 | 60.6 | 53.1 | 66.0 | *56.3* | < 0.001 |
| 50–59 | 31.0 | 33.3 | 25.6 | 31.1 | *27.4* | < 0.001 |
| 60–69 | 45.4 | 42.4 | 41.4 | 46.5 | *42.6* | |
| 70+ | 23.6 | 24.2 | 33.0 | 21.5 | *30.1* | |
| Medium/High Edu | 62.1 | 63.6 | 62.6 | 65.9 | *63.3* | 0.189 |
| Top wealth tertile | 22.8 | 39.2 | 37.5 | 39.0 | *37.2* | < 0.001 |
| Middle wealth tertile | 38.4 | 38.1 | 33.0 | 32.2 | *33.1* | |
| Lowest wealth tertile | 38.8 | 22.7 | 29.5 | 28.8 | *29.5* | |
| In work | 26.8 | 40.5 | 35.2 | 35.9 | *35.1* | < 0.001 |
| Voluntary work | 15.9 | 20.5 | 16.5 | 23.9 | *18.0* | < 0.001 |
| Fair/poor SRH | 25.7 | 14.8 | 23.7 | 17.7 | *22.3* | < 0.001 |
| Depressed | 13.4 | 10.6 | 11.4 | 11.4 | *11.5* | 0.744 |
| Disability | 28.9 | 18.9 | 21.1 | 16.3 | *20.4* | < 0.001 |
| Multimorbidity | 22.9 | 11.0 | 17.2 | 14.4 | *16.7* | < 0.001 |
| No children | 8.8 | 9.1 | 13.7 | 8.5 | *12.3* | < 0.001 |
| Children out HH | 77.1 | 75.0 | 76.1 | 80.2 | *76.8* | |
| 1 + Child in HH | 14.1 | 15.9 | 10.3 | 11.4 | *11.4* | |
| Has brothers/sisters alive | 77.5 | 84.1 | 81.2 | 81.9 | *81.3* | 0.217 |
| No parents alive | 70.4 | 65.5 | 80.0 | 64.6 | *76.1* | < 0.001 |
| Parent(s) aged < 85 | 11.6 | 17.8 | 9.9 | 16.0 | *11.5* | |
| 1 + parent aged 85+ | 18.0 | 16.7 | 10.1 | 19.4 | *12.4* | |
| Living alone | 3.4 | 10.5 | 25.4 | 14.3 | *21.9* | < 0.001 |
| Living with other/info missing | 9.0 | 9.0 | 8.6 | 9.8 | *8.8* | |
| Other in Fair/Poor Health * | 60.4 | 31.0 | 21.6 | 32.8 | *26.4* | < 0.001 |
| Other Depressed * | 26.6 | 9.0 | 6.8 | 10.3 | *8.7* | < 0.001 |
| Other Disabled * | 57.6 | 24.1 | 13.9 | 27.5 | *19.5* | < 0.001 |
| Other with Multimorbidity * | 29.9 | 16.6 | 13.0 | 16.3 | *14.7* | < 0.001 |
| Total Respondents - N | 284 | 264 | 4,797 | 1,216 | *6,561* | |

Source: English Longitudinal Study of Ageing (ELSA) Waves 6 (2012/13) – 9 (2018/19). P-value from chi-squared tests. Note: * These percentages are restricted to respondents living with another adult with available information.

Table 4 shows results from the multinomial logistic regression analyses regarding demographic, socioeconomic, health, and family factors measured at baseline as predictors of trajectories of informal care. Results suggest that women are more likely than men to be in the "stable intensive" (1.3 percentage point) or "decreasing" (7.6 percentage point) care trajectories, whereas they are 9.8 percentage points less likely to be in the "stable no care" group, after adjustment for other explanatory variables. People aged 70 and older at baseline are more likely to be in the "no care" trajectory and less likely to be in the "decreasing" or "stable intensive" ones than respondents in their 50s. There are also some socioeconomic differences across the trajectories of informal caregiving: for instance, those in the top wealth tertile distribution are more likely to be in the "stable no care" group and less likely to be in the "stable intensive care". Similarly, compared to those not in paid work, respondents in paid work are about 7 percentage points more likely to be in the "stable no care" group, but around 3 percentage points less likely to provide stable intensive care. Engagement in voluntary work also related to trajectories of caregiving, with those who volunteered in the month before the interview being 8.8 percentage points less likely to be in the “stable no care” group and 7.9 percentage points more likely to be in the “decreasing” care one. Those who rated their health as poor at baseline were more likely to fall in the “stable no care” group and less likely to be in any of the three remaining trajectories of caregiving. Finally, as the descriptives suggested, family composition and health are strongly related to trajectories of caregiving. People with children and parents alive are up to about 7 and 16 percentage points respectively less likely to be classified in the group “stable no care” compared to those without children and parents. Respondents with parents still alive are up to 12.5 percentage points more likely to be in the group of those whose caregiving commitment declines over time. Similarly, living with other people is generally associated with higher probabilities of being in one of the three trajectories of caregiving but the health profile of the adult respondents live with matters. For instance, if the adult they reside with is in poor self-rated health, respondents are 11.1 percentage points more likely to be in the “stable intensive” care category and 13.2 percentage points more likely to be in the “decreasing care” (suggesting that at baseline, living with someone with poor health increases dramatically the likelihood of providing care). However, if the health of the adult they live with is rated as good, the percentage points to fall in these two groups are much smaller (respectively 2.5 and 5.3). Very similar results and patterns are observed also when the co-residing adult’s disability, depression, and multimorbidity statuses are considered (see Supplementary Table 2 for details).

Table 4
Fully adjusted average marginal effects (with 95% CIs) for the relationship between demographic, socioeconomic, health, family characteristics and trajectories of informal caregiving

| | *Stable intensive care* | *Increasing intensive care* | *Stable no care* | *Decreasing care* |
|---|---|---|---|---|
| Female [a] | 1.26 * | 0.91 | -9.77*** | 7.59*** |
| | [0.28; 2.24] | [-0.07; 1.89] | [-11.9; -7.66] | [5.72; 9.46] |
| 60–69 [b] | -1.04 | 0.11 | 1.19 | -0.26 |
| | [-2.69; 0.60] | [-1.31; 1.53] | [-2.05; 4.44] | [-3.18; 2.66] |
| 70 + [b] | -2.50** | -0.02 | 8.28*** | -5.81*** |
| | [-4.28; -0.72] | [-1.80; 1.76] | [4.50; 12.1] | [-9.16; -2.48] |
| Medium/High education [c] | 0.35 | -0.47 | -0.53 | 0.65 |
| | [-0.67; 1.38] | [-1.55; 0.60] | [-2.78; 1.72] | [-1.36; 2.66] |
| Mid wealth tertile [d] | -0.57 | 1.35* | 0.37 | -1.15 |
| | [-1.99; 0.85] | [0.09; 2.60] | [-2.41; 3.16] | [-3.65; 1.34] |
| Top wealth tertile [d] | -2.92*** | 0.74 | 3.30* | -1.11 |
| | [-4.24; -1.60] | [-0.50; 1.98] | [0.46; 6.14] | [-3.69; 1.45] |
| In paid work [e] | -2.70*** | 0.04 | 7.25*** | -4.51*** |
| | [-3.83; -1.57] | [-1.26; 1.18] | [4.71; 9.78] | [-6.76; -2.25] |
| Voluntary work [f] | 0.27 | 0.64 | -8.78*** | 7.86*** |
| | [-1.12; 1.66] | [-0.69; 1.99] | [-11.7; -5.87] | [5.16; 10.6] |
| Depressed [g] | -0.88 | 0.40 | -0.96 | 1.44 |
| | [-2.24; 0.48] | [-1.36; 2.16] | [-4.51; 2.58] | [-1.78; 4.67] |
| Fair/poor SRH [h] | -1.25* | -1.57* | 6.68*** | -3.86** |
| | [-2.42; -0.08] | [-2.76; -0.38] | [3.91; 9.44] | [-6.35; -1.36] |
| Disability [i] | 1.64* | 0.99 | 0.37 | -3.00* |
| | [0.18; 3.10] | [-0.58; 2.56] | [-2.56; 3.30] | [-5.52; -0.47] |
| Multimorbidity [j] | 1.41 | -1.00 | -0.64 | 0.23 |
| | [-0.07; 2.89] | [-2.32; 0.32] | [-3.69; 2.40] | [-2.54; 3.01] |
| Child(ren) live out HH [k] | 2.13 | 0.56 | -6.82*** | 6.04*** |
| | [-1.43; 1.86] | [-0.92; 2.04] | [-9.97; -3.65] | [3.34; 8.73] |
| 1 + Child(ren) in HH [k] | 1.35 | 1.54 | -6.01** | 3.12 |
| | [-0.88; 3.59] | [-0.54; 3.62] | [-10.3; -1.73] | [-0.50; 6.73] |
| Has brothers/sisters [l] | -2.04** | 0.36 | 3.15* | -1.46 |
| | [-3.56; -0.52] | [-0.90; 1.62] | [0.33; 5.95] | [-4.00; 1.07] |
| Parent(s) < 85 [m] | 0.34 | 2.14* | -11.7*** | 9.27*** |
| | [-1.39; 2.07] | [0.05; 4.23] | [-15.9; -7.59] | [5.43; 13.1] |
| Parent(s) > = 85 [m] | 2.39* | 1.52 | -16.4*** | 12.5*** |
| | [0.55; 4.24] | [-0.16; 3.19] | [-20.0; -12.8] | [9.15; 15.9] |
| With adult: good SRH [n] | 2.54*** | 2.08*** | -9.90*** | 5.27*** |
| | [1.78; 3.29] | [0.97; 3.20] | [-12.5; -7.31] | [2.91; 7.63] |
| With adult: poor SRH [n] | 11.1*** | 3.60*** | -27.9*** | 13.2*** |
| | [9.32; 13.0] | [2.01; 5.18] | [-31.3; -24.5] | [10.1; 16.2] |
| With adult (missing) [n] | 3.28*** | 1.54 | -10.6*** | 5.81** |
| | [1.61; 4.95] | [-0.35; 3.43] | [-15.0; -6.28] | [1.91; 9.72] |

Source: English Longitudinal Study of Ageing (ELSA) Waves 6 (2012/13) – 9 (2018/19). N = 6,424. Values in brackets show the 95% CIs. *P < 0.05; **p < 0.01; ***p < 0.001. Notes: HH = household; SRH = Self-Rated Health. Reference categories are a) Male; b)50–59; c) Low education; d) Low wealth tertile; e) Not in paid work; f) No voluntary work; g) Not depressed; h) At least good SRH; i) No disability; j) No multimorbidity; k) No children; l) No brothers or sisters alive; m) No parents alive; n) Lives alone.

Finally, Table 5 shows results from the multinomial logistic regression analyses regarding the associations between changes in selected family and health compositions and trajectories of informal caregiving. Once again, the findings are reported as AMEs; per cent distributions of each variable are available in Supplementary Table 3. Overall, there is little evidence of an association between changes in personal health and those trajectories of caregiving that also show changes in the predicted probabilities of providing informal care over time. However, changes in family characteristics are significantly associated also with changes in care over time. For instance, respondents who lost their parents during the years under study are 12 percentage points respectively more likely to be classified in the group “decreasing care” compared to those without parents or whose parents are still alive. Similarly, respondents whose household composition changed and who lived alone by wave 9 (and who have mostly become widowed) are 3 percentage points less likely to be in the “increasing intensive care” group but almost 15 percentage points more likely to be in the “decreasing care” group. Similarly, when changes to the health profile of the adults respondents live with are taken into account, results suggest, unsurprisingly, that the deterioration in the physical health of the co-residing adult is associated with higher percentages of belonging to the “increasing intensive care” group, whereas if the co-residing adult’s health improves respondents are more likely to the decreasing care trajectory.

Table 5
Fully adjusted average marginal effects (with 95% CIs) for the relationship between changes in selected health and family characteristics between wave 6 and wave 9 and trajectories of informal caregiving

| | *Stable intensive care* | *Increasing intensive care* | *Stable no care* | *Decreasing care* |
|---|---|---|---|---|
| No changes in depression | *Ref* | | | |
| Has become depressed | *3.21***<br>*[0.74; 5.69]* | *0.02*<br>*[-1.92; 1.97]* | *-5.38**<br>*[-9.90; -0.86]* | *1.91*<br>*[-2.01; 5.83]* |
| No longer depressed | *1.33*<br>*[-0.87; 3.54]* | *0.61*<br>*[-1.47; 2.71]* | *-1.58*<br>*[-5.99; 2.82]* | *0.37*<br>*[-4.14; 3.40]* |
| No changes in SRH | Ref | | | |
| Has become with poor SRH | 0.93<br>[-0.80; 2.67] | -0.65<br>[-2.14; 0.84] | -2.77<br>[-6.33; 0.77] | 2.49<br>[-0.69; 5.67] |
| No longer with poor SRH | -0.07<br>[-2.01; 1.86] | -1.63<br>[-3.39; 0.12] | 3.53<br>[-0.66; 7.73] | -1.82<br>[-5.53; 1.89] |
| No changes in disability | ref | | | |
| Has become disabled | 0.54<br>[-0.97; 2.07] | -1.19<br>[-2.52; 0.14] | 0.80<br>[-2.34; 3.96] | -0.06<br>[-2.88; 2.75] |
| No longer disabled | 3.27*<br>[0.77; 5.78] | 2.04<br>[-0.34; 4.42] | -7.62**<br>[-12.2; -2.98] | 2.30<br>[-1.68; 6.29] |
| No changes in multimorbidity | ref | | | |
| Has reported multimorbidity | 0.19<br>[-1.57; 1.96] | -0.47<br>[-2.07; 1.12] | 0.68<br>[-3.03; 4.39] | -0.40<br>[-3.69; 2.89] |
| No longer with multimorbidity | 0.96<br>[-2.09; 4.03] | 0.21<br>[-2.56; 2.99] | 1.51<br>[-4.42; 7.44] | -2.69<br>[-7.73; 2.34] |
| Parent(s) died | 1.19<br>[-0.41; 2.80] | 0.16<br>[-1.26; 1.60] | -12.9***<br>[-16.4; 9.4] | 11.6***<br>[8.32; 14.7] |
| No co-resident health changes | ref | | | |
| Lives alone at wave 9 | -3.04***<br>[-4.27; -1.81] | -2.82***<br>[-4.05; -1.61] | -9.48***<br>[-14.1; -4.87] | 15.4***<br>[10.8; 19.8] |
| With adult: poor SRH at wave 9 | 2.43*<br>[0.20; 4.66] | 4.51***<br>[2.12; 6.90] | -12.0***<br>[-15.2; 6.77] | 3.34<br>[-1.09; 7.78] |
| With adult: no longer in poor SRH at wave9 | -0.11<br>[-2.35; 2.11] | 1.39<br>[-1.05; 3.84] | -4.61<br>[-9.67; 0.43] | 5.06**<br>[1.41; 8.71] |
| With adult: disabled at wave 9 | 3.58**<br>[1.46; 6.24] | 6.71***<br>[4.06; 9.38] | -12.2***<br>[-16.4; -7.92] | 1.60<br>[-1.95; 5.16] |
| With adult: no longer disabled at wave 9 | -1.00<br>[-3.07; 1.07] | 1.36<br>[-1.12; 3.85] | -6.02<br>[] | 5.66*<br>[0.89; 10.4] |
| With adult: multimorbidity at w9 | 2.81*<br>[0.21; 5.42] | 3.23*<br>[0.46; 6.01] | -8.77***<br>[-13.7; -3.85] | 2.72<br>[-1.43; 6.89] |
| With adult: no longer multimorbidity at wave 9 | 1.38<br>[-2.07; 4.83] | 3.04<br>[-0.81; 6.98] | -5.35<br>[-12.2; 14.6] | 1.35<br>[-5.47; 5.74] |

Source: English Longitudinal Study of Ageing (ELSA) Waves 6 (2012/13) – 9 (2018/19). Notes: Changes are obtained by comparing characteristics at wave 6 and wave 9. Those who "became unhealthy" are respondents who reported the health condition at wave 9 but not at wave 6. On the other hand, those who reported health conditions at wave 6 but not at wave 9 are classified as "no longer unhealthy". The same principle applies to changes in household compositions and to the health of the co-residing adult. All sets of multinomial logistic regressions adjusted for gender, age groups, education, and wealth at wave 6.

## 4. Discussion

In the context of an ageing population, combined with long-standing challenges in the delivery of formal social care for older people, unpaid caregivers play a key role in promoting the quality of life of older people and their extended families and ensuring that needs for care and support are met. Although the provision of informal care is often a process, most studies have provided snapshots of caregiving overlooking its dynamic nature. Using data spanning 6 years from the nationally representative ELSA, we aimed to describe trajectories of caregiving in later life and the factors associated with them.

Overall, we found four distinct trajectories of informal caregiving with two third of the sample under study never engaging in care provision throughout the 6 years under study, 5% providing intensive care throughout, and the remaining 30% showing a decreasing (23%) and increasing (7%) trajectory of informal care provision. These results show heterogeneity and complexity in the provision of informal care in later life, as reported also in studies conducted in Australia and The Netherlands (Tooth and Mishra 2014; Verbakel and Glijn 2023). However, the number and prevalence of trajectories of caregiving in those studies are slightly different – this might be influenced not only by the measures and operationalisations of informal care provisions in the study, but also by external conditions (including formal care provision, generational differences in attitudes towards informal care, and employment policies) that can facilitate or restrict the provision of care in later life (Albertini and Kohli 2013; Price et al. 2018; van Damme and Spijker 2024).

This study also investigated the links between demographic, socio-economic, health, and family indicators and trajectories of caregiving. It is often argued that the onset of informal care provision and the subsequent commitment to this activity are not random but depend broadly on three factors including the care receiver's need for care, individual predisposing and enabling factors (including gender, health, and wealth), and the context (Brandt et al. 2009; Broese van Groenou and De Boer 2016). For instance, for socioeconomic factors, many scholars argue that people with fewer resources and time constraints might have less to "lose" by becoming carers compared to those in employment or better off who have the resources to access, purchase, and use alternative forms of care, help, and support from the market (de Zwart et al. 2017; Di Gessa et al. 2022; Quashie et al. 2022; Saito et al. 2018). It is often also suggested that family caregiving remains a predominantly "feminine" activity because of the gendered nature of different tasks, expectations of behaviours, responsibilities, and social structures and norms (Haberkern et al. 2015; Sharma et al. 2016).

In our study, we also found that some of the personal demographic and socioeconomic characteristics were related to caregiving trajectories. For instance, in line with previous cross-sectional and longitudinal studies, women were generally more likely to belong to a caregiving trajectory, and older respondents were more likely to be in the "stable no care" one. Also, our results indicate that people in poorer socioeconomic status (in the lowest wealth tertile and not in paid work) were more likely to provide care intensively throughout, as found also in Tooth and Mishra (2014). We also found that health factors were associated with trajectories of caregiving: those in good self-rated health at baseline were generally more likely to provide care, with some indication though that those with disabilities were related to the "stable intensive" care group. However, changes in personal health were not associated with changing trajectories of care provision. This could reflect both a selection and a consequence of these trajectories of informal caregiving, with care providers often with a poorer health profile than non-carers but better than that of the person they cared for (Price and Di Gessa 2023).

Overall, though, our results show that "needs" factors – operationalised in this study with the availability and health of family members – were collectively the most immediate and strongly associated with trajectories of caregiving. This is in line with all models that position family care provision as stemming from having a close kin or friend who needs care (Brandt and Deindl 2017; Broese van Groenou and De Boer 2016). Although the availability of parents and spouses as well as their health progression (and therefore of their needs and demands) are the main theoretical drivers of onset and changes in informal care provision in later life, studies have often overlooked this aspect. In our study, we found that older people who live alone, with no children, and no parents alive are more likely to never provide care, whereas those with older parents and those who live with adults in poor health are more likely to provide stable intensive care. Also, our results suggest that changes in trajectories of caregiving are mostly related to changes in family circumstances. For instance, older people who experienced losses of parents and/or partners, were most likely to belong to the "decreasing" caregiving trajectory whereas those whose partner's health deteriorates over time are significantly associated with the "increasing" trajectory of informal care provision.

### 4.1 Strengths and limitations

We described trajectories of caregiving by older English people over 6 years, and sociodemographic, health, and family factors associated with these trajectories. To our knowledge, this was the first study to investigate this issue using a large scale nationally representative prospective survey that did not rely on the retrospective recollection of care and that accounted for a wide range of family characteristics, including the presence of parents and children, and the health of cohabiting adults. Our study demonstrates that care provision evolves over time and "needs" factors are most likely to relate to trajectories of informal caregiving, highlighting the limitations of a knowledge base founded on single care episodes.

Our analyses, however, also have some limitations. ELSA does not collect detailed information about the care provided to each recipient but rather asks generic questions (related to all recipients of care) and the time spent altogether looking after them. Therefore, in our trajectories and particularly for those who care for more than one person, we could not distinguish between different intensities of care or focus on specific care recipients. Moreover, we lack detailed information on the recipient of care: except for (the majority of) those who look after their cohabiting spouse/partner, we do not know for instance where the recipients of care live or their health status. Also, the caregiver-care recipient relationship, information on whether anyone else is involved in the provision of care (including other family members or friends as well as formal care providers), personal preferences for informal care (provision and receipts), and personality factors are all missing and would be useful to better describe and distinguish trajectories of caregiving. More generally, ELSA is also lacking information on the broad domains and multiple tasks and activities that characterize family caregiving (that range from assistance with daily activities and providing direct care to the recipient to navigating complex health care and giving emotional or practical help with paperwork). Also, although some information on the intensity of care is provided, most information refers to care provided in the week prior to the interview with little understanding of whether this was a one-off or more regular commitment. Similarly, 2-yearly surveys might miss more sporadic caregiving trajectories. Finally, although other studies in

The Netherlands and Australia have found similar patterns of caregiving (Tooth and Mishra 2014; Verbakel and Glijn 2023), we acknowledge that the trajectories found in our study and factors associated with them may vary across countries with different formal long-term care settings or family-norms. Moving forward, studies should also assess whether trajectories of caregiving differ across different cohorts and to what extent they relate to long-term health trajectories as both issues were beyond the scope of this study.

## 4.2 Conclusion

To conclude, our study shows that providing informal care in later life is a dynamic process, with one in 20 older people providing intensive care throughout a period of 6 years and 30% changing their probability to provide informal care, with both increasing and decreasing caregiving experiences over time. Although personal sociodemographic and health characteristics are useful factors associated with trajectories of informal caregiving, our results suggest that it is the availability of family (and potentially friends) and their needs and health profiles that are the main drivers that shape trajectories of informal care in later life. However, future research should aim to further investigate whether and to what extent trajectories of caregiving could be qualitatively distinct depending on the recipients' specific health characteristics, the relationship with the recipient, and more broadly arrangements with other family members or friends or other commitments including grandchild care provision or employment.

### Declarations

**Statements and Declarations**

**Funding:** The English Longitudinal Study of Ageing is funded by the National Institute on Aging (R01AG017644), and by UK Government Departments coordinated by the National Institute for Health and Care Research (NIHR). This study was supported by funding within the Joint Programming Initiative More Years, Better Life from the following national funding bodies: UK Economic and Social Research Council (ES/W001454/1) and the Federal Ministry of Education and Research, Germany (BMBF) (Grant Number: 16SV8702).

**Conflict of interest:** The authors have no relevant financial or non-financial interests to disclose.

**Availability of data and material:** Researchers can download all waves of ELSA data from the UK Data Service (SN: 5050, DOI: http://doi.org/10.5255/UKDA-SN-5050-27). For more information on how to access ELSA visit https://www.elsa-project.ac.uk/accessing-elsa-data

**Code availability**: All analyses were performed using Mplus and Stata.

**Authors' contribution:** G Di Gessa conducted analyses, drafted the paper, and co-led the interpretation of data with C Deindl. C Deindl and G Di Gessa led the conception and design of the study. Both authors contributed to the interpretation of data and to manuscript revisions. All authors have read and approved the final version.

**Ethics Approval:** ELSA was approved by the London Multicentre Research Ethics Committee (MREC/01/2/91).

**Consent to participate**: Informed consent was obtained from all ELSA participants.

### Figures

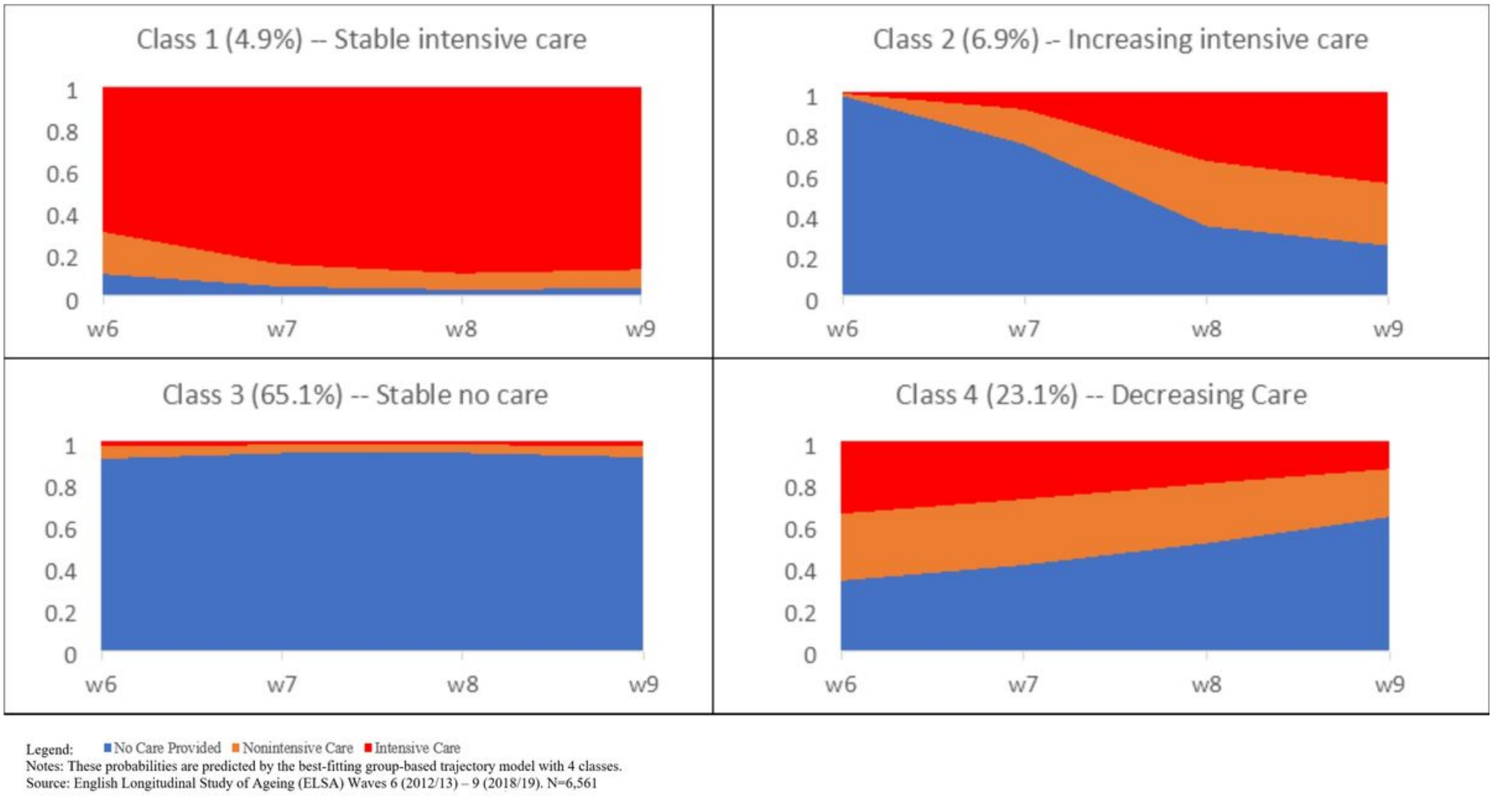

**Figure 1**

**Stacked predicted probabilities of informal care provision(“No care”; “Non-intensive care”; and “Intensive care”)**

### Supplementary Files

This is a list of supplementary files associated with this preprint. Click to download.

- EJASupplementaryMaterial.docx
